# BlueRecording: A pipeline for the efficient calculation of extracellular recordings in large-scale neural circuit models

**DOI:** 10.1371/journal.pcbi.1013023

**Published:** 2025-05-23

**Authors:** Joseph James Tharayil, Jorge Blanco Alonso, Silvia Farcito, Bryn Lloyd, Armando Romani, Elvis Boci, Antonino Cassara, Felix Schürmann, Esra Neufeld, Niels Kuster, Michael Reimann

**Affiliations:** 1 Blue Brain Project, École polytechnique fédérale de Lausanne (EPFL) Campus Biotech, Geneva, Switzerland; 2 Foundation for Research on Information Technologies in Society (IT’IS), Zurich, Switzerland; Centre National de la Recherche Scientifique, FRANCE

## Abstract

As the size and complexity of network simulations accessible to computational neuroscience grows, new avenues open for research into extracellularly recorded electric signals. Biophysically detailed simulations permit the identification of the biological origins of the different components of recorded signals, the evaluation of signal sensitivity to different anatomical, physiological, and geometric factors, and selection of recording parameters to maximize the signal information content. Simultaneously, virtual extracellular signals produced by these networks may become important metrics for neuro-simulation validation. To enable efficient calculation of extracellular signals from large neural network simulations, we have developed *BlueRecording*, a pipeline consisting of standalone Python code, along with extensions to the Neurodamus simulation control application, the CoreNEURON computation engine, and the SONATA data format, to permit online calculation of such signals. In particular, we implement a general form of the reciprocity theorem, which is capable of handling non-dipolar current sources, such as may be found in long axons and recordings close to the current source, as well as complex tissue anatomy, dielectric heterogeneity, and electrode geometries. To our knowledge, this is the first application of this generalized (i.e., non-dipolar) reciprocity-based approach to simulate EEG recordings. We use these tools to calculate extracellular signals from an *in silico* model of the rat somatosensory cortex and hippocampus and to study signal contribution differences between regions and cell types.

## Author summary

In this paper, we introduce *BlueRecording*, a suite of software tools that enables the simulation of extracellular recordings from large-scale neural circuit models. With BlueRecording, users can simulate EEG, ECoG, and intracellular recordings, using a variety of signal estimation methods. Notably, BlueRecording implements the innovative “generalized reciprocity approach”, which allows the accurate calculation of signals with arbitrarily complex electrode shapes and arrangements and heterogeneous, anisotropic head models. BlueRecording is tightly integrated into the CoreNEURON simulation engine, leading to a 3-fold improvement in computation time compared to the state-of-the-art. It is compatible with the SONATA format for neural circuit models; as demonstrators, we compute signals from *in silico* models of the rat somatosensory cortex and of the hippocampus using BlueRecording, which permits us to disambiguate the contributions of different cell types to the EEG signal.

## 1. Introduction

The source of key signals measured in neuroscience is the transmembrane current generated by the electrical activity of neurons. Computational investigation of such signals using compartmental models requires: (1) a model and simulator that calculate the membrane currents, and (2) an estimation of how each compartment’s current affects the signal. For (2), typically simplifying approaches have been used. Simply comparing model output to reconstructed current dipoles relies on accurate and reliable source reconstruction from EEG data using inverse solution methods [1] – something that is difficult to achieve because of the poorly conditioned inverse problem and the complex impact of volume conduction in the heterogeneous head anatomy on EEG signal formation. Point-source and line-source approaches approximate the properties of the different tissues as homogeneous [2–4]. They are therefore only valid where electrodes are within the brain tissue, where some degree of homogeneity of the nearby dielectric environment can be assumed. The commonly employed simplified variant of the reciprocity theorem approximates individual neurons or groups of neurons as dipolar sources. It is therefore only valid when electrodes are sufficiently far away from said neurons [5].

Our goals in this work were to: provide a solution to (2) that is equally valid for both proximal and distal electrical measurements; provide flexibility to researchers with respect to the approaches to both (1) and (2) they want to employ; and to support very large electrophysiological models by enabling online and efficient computation.

We use the general form of the reciprocity theorem developed by Plonsey [6], which supports full compartmental resolution of the neuronal models and takes into account the dielectric properties of all tissues, to calculate the impact of each compartment on the signal. It can be employed for EEG, ECoG and LFP, and is capable of dealing with arbitrarily shaped electrode geometries. The impact of each compartment on the signal is stored in what we call a weights file. We then implemented extensions to the Neurodamus simulation control software [7] and the CoreNEURON simulation engine [8] to consume a weights file to calculate the signal during a simulation run and store the result for later analysis. Together, we call the applications to calculate weights files and the extensions to the simulator the BlueRecording framework.

The formalism of a standardized weights file ensures flexibility with respect to objective (2). A user can easily implement a different solution so long as it provides a valid weights file. Since large-scale simulations are required for the EEG signal, the ability to use the highly efficient CoreNEURON simulation engine regardless of dielectric model is a significant advantage. In addition, BlueRecording is compatible with any circuit defined in the SONATA format [9], making it easily portable between different simulators, and therefore highly flexible with respect to objective (1) as well.

In this paper, we apply BlueRecording to microcircuit models developed by the Blue Brain Project (BBP). The BBP somatosensory cortex model consists of ~4.2 million reconstructed neurons with accurate morphologies, optimized physiological properties, and algorithmically generated connectivity [10]. The BBP hippocampus CA1 model consists of ~456,000 mophologically detailed neurons, with properties and connectivity generated in a similar manner to [10], along with virtual innervation by Schafer collaterals and cholinergic modulation [11]. The support for spatially extended and morphologically highly detailed BBP microcircuits is a particular advantage when studying EEG signals. This is demonstrated here by comparing virtual LFP, EEG, and ECoG, and identifying various contributions to the signals.

## 2. Design and implementation

The extracellular signals are calculated online in the NEURON simulation environment. Due to the linear nature of Maxwell’s equation, the signal at a particular electrode (relative to another electrode or ground) is amenable to the form

$$V(t)=\Sigma_{i=1}^{m}\Sigma_{j=1}^{n}C_{i,j}{\text{i\_membrane}}_{i,j}(t)$$ (1)

where i\_membrane is the total compartmental transmembrane current (i.e., the sum of the ionic and capacitive currents, as described in [2]), *i* is the index of each neuron in the circuit, *j* is the index of each neural compartment in the neuron, and *C* is a scaling factor (‘weight’), the calculation of which is discussed below. The equation takes the form of the commonly employed lead-field matrices [12], which are frequently applied to dipole source distributions, but have also been applied directly to transmembrane currents [13–17]. The simulator supports simultaneous recording from an arbitrary number of electrodes.

### 2.1. Calculation of coefficients

#### 2.1.1. Generalized reciprocity approach.

BlueRecording permits the calculation of extracellular signals using the reciprocity theorem [6]. The reciprocity theorem establishes a simple relationship between the electric field induced at different locations in the brain by applying a current through two surface electrodes, and the voltage difference measured between these electrodes when placing a current source at the same brain locations. Intuitively, if a source at location A produces a voltage at location B, a source at location B will produce an identical voltage at location A – hence the term “reciprocity”. As a result, measurable signals from distributed and dynamic brain activity can be computed using a single electromagnetic simulation, rather than needing a distinct simulation per independent source activity component.

More formally, following Plonsey [6],let us suppose that we have a a volume current source *I*_*U*_(*t*) in an arbitrary dielectric environment *U* (in the case of a neural current source, *I*_*U*_(*t*) is the set of neural segment currents $\left\{{\text{i\_membrane}}_{i,j}(t)\;\forall \;i,j\right\}$, where *i* is the index of each neuron and *j* is the index of each segment within a neuron). Further, let $\Phi_{1}(t)$ be the potential field resulting from that current source *I*_*U*_(*t*). Now, suppose that instead of a volume current source, we have a surface current distribution *K* on the surface of the environment *U*. Let $\Phi_{2}(t)$ be the potential field resulting from *K*.

It can be shown (for a full derivation see [6]) that ${∰}_{U}I_{U}(t)\cdot \Phi_{2}dU={∯}_{S}\Phi_{1}(t)\cdot KdS$.

When *K* consists of a pair (*a*,*b*) of quasi-perfect electric conductors (e.g., metallic EEG electrodes) providing a (virtual) current *J*, the situation simplifies to ${∰}_{U}I_{U}(t)$ · $\Phi_{2}dU=J$ · $\left(\Phi_{1,a}-\Phi_{1,b})=J\cdot V(t\right)$, where $V(t)=\Phi_{1,a}1(t)-\Phi_{1,b}$ corresponds to the measured signal.

For a neurite with electrodes sufficiently far away, we can treat each nodal current ${\text{i\textbackslash{}\_membrane}}_{i,j}(t)\in I_{U}(t)$ as point current sources at points ${\vec{r}}_{1}...{\vec{r}}_{k}$, respectively. Thus, the left side of the equation reduces to $\Sigma_{i=1}^{m}\Sigma_{j=1}^{n}{\text{i\textbackslash{}\_membrane}}_{i,j}(t)\Phi_{2}({\vec{r}}_{i,j})$ and the potential between the two electrodes in this situation becomes

$$V(t)=\Phi_{1}({\vec{r}}_{a})-\Phi_{1}({\vec{r}}_{b})=\frac{\Sigma i=1^{m}\Sigma_{j=1}^{n}i\_membrane\_i,j(t)\Phi_{2}({\vec{r}}_{i,j})}{J}$$ (2)

The coefficients *C*_*j*_ used to calculate the extracellular signal as described in Eq 1 are therefore given by

$$C_{i,j}=\frac{\Phi_{2}({\vec{r}}_{i,j})}{J}$$ (3)

In order to calculate $\Phi_{2}({\vec{r}}_{i,j})$, a finite element method (FEM) simulation of a head model with a recording electrode array is performed, where a (virtual) current is applied between a pair of recording electrodes, and the potential field is interpolated at the locations of the neural segments ${\vec{r}}_{i,j}$. Information about the FEM rat head model setups created for this study is provided in S1 Methods.

#### 2.1.2. Dipole-based reciprocity approach.

If, for each neuron *i*, we take the Taylor series expansion of $\Phi_{2}({\vec{r}}_{i,j})$ around the vector ${\hat{r}}_{i}$ – the geometric center of the neuron – we obtain, to first order

$$\Phi_{2}({\vec{r}}_{i,j})\approx \Phi_{2}({\hat{r}}_{i})+\nabla \Phi_{2}\cdot ({\vec{r}}_{i,j}-{\hat{r}}_{i})$$ (4)

Substitution Eq 4 into Eq 2 and rearranging, we obtain

$$J\cdot V(t)\approx \Sigma_{i=1}^{m}[\Phi_{2}({\hat{r}}_{i})\Sigma_{j}{\text{i\_membrane}}_{i,j}(t)+\Sigma_{j}{\text{i\_membrane}}_{i,j}(t)\nabla \Phi_{2}\cdot ({\vec{r}}_{j}-{\hat{r}}_{i})]$$ (5)

For a neural current source, $\Sigma_{j}{\text{i\_membrane}}_{i,j}=0$ by Kirchhoff’s Law. Thus,

$$V(t)\approx \Sigma_{i}\frac{\nabla \Phi_{2}\cdot {\vec{p}}_{i}(t)}{J}=-\Sigma_{i}{\vec{E}}_{n}({\hat{r}}_{i})\cdot {\vec{p}}_{i}(t)$$ (6)

where ${\vec{p}}_{i}(t)$ is the current dipole calculated for neuron *i* at each time step as

$${\vec{p}}_{i}(t)=\Sigma_{j}{\text{i\_membrane}}_{i,j}(t)\cdot ({\vec{r}}_{i,j}-{\hat{r}}_{i}).$$ (7)

The normalized ${\vec{E}}_{n}=-\nabla \Phi_{2}/J$ is the ‘lead-field’. ${\vec{E}}_{n}$ can be computed using a FEM model, as described in S1 Methods, and is interpolated at the geometric center of each neuron, ${\hat{r}}_{i}$. Substituting into Eq 1 and rearranging, we find that

$$C_{i,j}=-{\vec{E}}_{n}({\hat{r}}_{i})\cdot ({\vec{r}}_{i,j}-{\hat{r}}_{i}).$$ (8)

While the choice of ${\hat{r}}_{i}$ is not relevant from an analytical perspective, it can be numerically advantageous to place it near the neuron center.

#### 2.1.3. Calculation of coefficients with line-source approximation.

Unlike EEG or ECoG electrodes, LFP electrodes are small and located within the cortical tissue. If the LFP electrode can be treated as point-like, the tissue within the relevant environment is homogeneous and isotropic, and the ground reference sufficiently distant to be treated as being at infinity and omnidirectional, the computation of an ‘exposing’ potential required by the reciprocity theorem can be avoided and instead the line-source approximation can be used to compute the coefficients for LFP calculations without need for FEM simulation.

According to the line-source approximation [18], the contribution to the electric potential at a point electrode location of a neural segment situated in a homogeneous, isotropic environment is given by $\Phi_{i,j}=\frac{1}{4\pi \sigma_{e}\Delta_{s}}\int_{-\Delta_{s}}^{0}\frac{{\text{i\_membrane}}_{i,j}(t)\cdot ds}{\sqrt{(h-s)2+r^{2}}}$, where $\Delta_{s}$ is the length of the segment, *h* is the distance from the near end of the segment to the recording electrode in the axial direction, and *r* is the absolute value of the distance from the near end of the segment to the electrode in the perpendicular direction. Evaluating the integral analytically and substituting into Eq 1, we find that:

$$C_{i,j}=\frac{1}{4\pi \sigma_{e}\Delta_{s}}\left\{ \begin{array}{cc} log(\frac{\sqrt{h^{2}+r^{2}}-h}{\sqrt{l^{2}+r^{2}}-l}) & h<0,l<0\\ log(\frac{\left(\sqrt{h^{2}+r^{2}}-h)(l+\sqrt{l^{2}+r^{2}}\right)}{r^{2}} & h<0,l>0\\ log(\frac{\sqrt{l^{2}+r^{2}}+l}{\sqrt{h^{2}+r^{2}}+h}) & h>0,l>0 \end{array}\right..$$ (9)

With $\left(x_{1},y_{1},z_{1}\right)$ and $\left(x_{2},y_{2},z_{2}\right)$ as the endpoints of the segment and (*x*,*y*,*z*) as the location of the electrode,

$$h=\frac{1}{\Delta_{s}}(x-x_{2},y-y_{2},z-z_{2})\cdot (x_{2}-x_{1},y_{2}-y_{1},z_{2}-z_{1})$$ (10)

$$r^{2}=(x-x_{2}{)}^{2}+(y-y_{2}{)}^{2}+(z-z_{2}{)}^{2}-h^{2}$$ (11)

$$l=h+\Delta_{s}$$ (12)

#### 2.1.4. Calculation of coefficients with point-source approximation.

A further simplification can be made (in addition to the assumptions of an infinite homogeneous medium, a point-like electrode, and reference at infinity) by treating each neural compartment as a point rather than a line. In this case, the potential at the recording electrode is given by

$$V=\frac{1}{4\pi \sigma_{e}}\Sigma_{i}\Sigma_{j}\frac{{\text{i\_membrane}}_{i,j}(t)}{\left|{\vec{r}}_{i,j}-{\vec{r}}_{e}\right|}$$ (13)

where ${\vec{r}}_{e}$ is the position of the electrode and ${\vec{r}}_{i,j}$ is the position of the compartment. Substituting into Eq 1 we obtain:

$$C_{i,j}=\frac{1}{4\pi \sigma_{e}|{\vec{r}}_{i,j}-{\vec{r}}_{e}|}$$ (14)

#### 2.1.5. Choice of voltage and spatial reference.

The extracellular signals produced using the reciprocity approaches are gauge-independent with respect to the potential field (i.e., the location to which a potential of 0V is assigned is arbitrary). This is because the sum of membrane currents of the compartments over a neuron must be zero. Thus, any constant added to the compartment weights does not affect the signal, and only the difference in compartment weights matters. For better visualization, we can therefore add an offset to the compartment weights in each neuron such that the minimum weight per neuron is 0.

#### 2.1.6. Numerical considerations.

Calculation of the extracellular signal involves summation over a large number of terms that typically compensate, potentially leading to important numerical errors as a large number of digits can be significant. Because of gauge freedom and current conservation, this can be mitigated by selecting good reference values for ${\hat{r}}_{i}$ and $\Phi_{2}$, and if that proves insufficient, by using a compensation algorithm such as Kahan summation. Our implementation does not use Kahan summation, as we have not found it to be necessary for the current study. However, awareness that compensated summation might be required in other situations, is necessary.

### 2.2. Workflow

**Fig 1:**
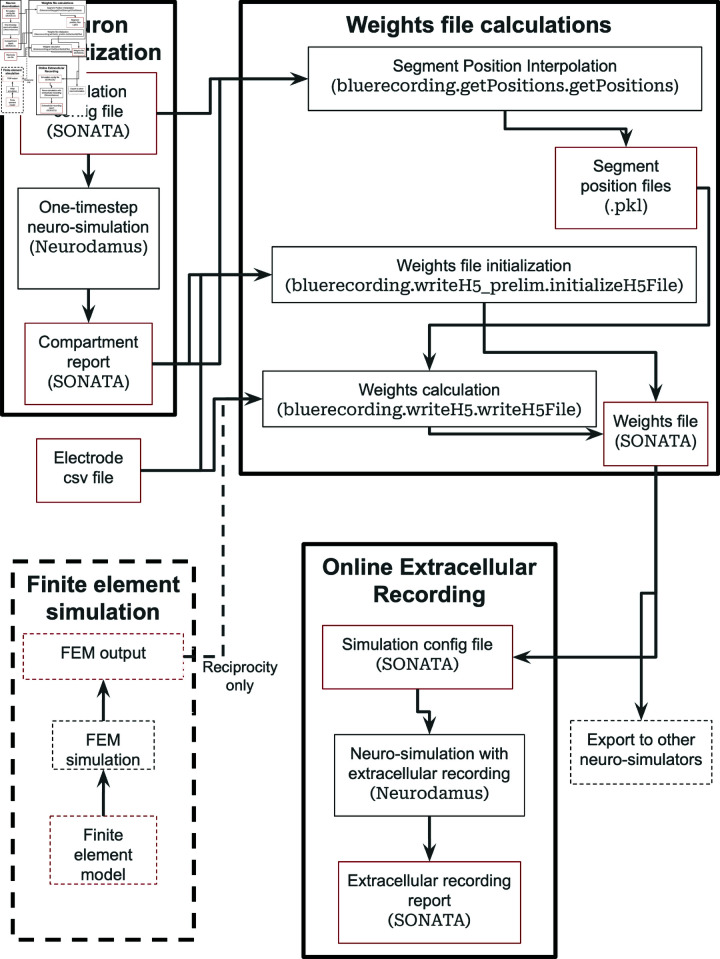
Workflow for the BlueRecording Pipeline. Input and output files are shown in red boxes, while processes are shown in black boxes. Dashed lines around the FEM simulation step indicate that it is only used for reciprocity-based calculations

The workflow for calculating coefficients and running an online extracellular recording simulation with BlueRecording is as follows (see as well Fig 1). Tools used for each step are listed in parentheses:

1. **Calculate Segment Positions:** Segment positions are calculated in two steps
  - **Run Neuro-Simulation (Neurodamus):** A minimal (at least one time step) neuro-simulation is run to produce a compartment report that reveals how the neurons are discretized.
  - **Interpolate segment positions (bluerecording.getPositions. getPositions()):** The compartment report and information from the simulation configuration file are used to interpolate the segment positions from the 3d coordinates in the morphology file, which are not necessarily aligned to the discretized compartments
2. **Create electrode csv file (Manual)** See Sect 2.3.2
3. **Initialize Electrode Weights file (bluerecording.writeH5 _prelim.initializeH5File()):** This step reads metadata from the electrode csv file and the discretization from the compartment report, and writes both to the weights file. See Sect 2.3.1 for details on the file format and 2.3.3 for details on writing the file
4. **Calculate weights:**
  - **Reciprocity-based methods**
    (i) **FEM simulations (Any FEM solver):** A FEM simulation is run to calculate the potential field generated by running a current between a recording electrode and a reference electrode. The results (see S1 Table) are exported.
    (ii) **Interpolation of weights (bluerecording.writeH5.writeH5File()):** From the exported results, the weights are calculated by interpolating the potential field at the neural compartment locations (for the generalized reciprocity approach) or by taking the scalar product of the E field at the center of the neuron and the segment position vectors (for the dipole-reciprocity approach).
  - **Analytic calculations (bluerecording.writeH5.writeH5File()):** For the line-source and point-source approximations, the weights are calculated analytically, without requiring FEM simulations.
5. **Run neuro-simulations (Neurodamus or alternative neuro-simulator)** While it is intended that simulations be run using Neurodamus as described in Sect 2.3.4, other simulators may be extended to also support the weights file created in BlueRecording.

### 2.3. Implementation details

#### 2.3.1. Extension to SONATA-format.

We defined an extension to the SONATA format [9] to store the coefficients *C*_*i*,*j*_. Briefly, the SONATA format treats each neuron in the network as a **node**, and nodes are organized into **populations**. The coefficients are stored in an HDF5 file (Table 1), which also includes metadata on the type and location (both in Cartesian space and with respect to the anatomy) for each electrode. For each population, the coefficients for all nodes and all electrodes are listed in a single array; for each node, the coefficients (one per compartment) are listed consecutively. For each population, there is also a dataset listing the IDs of each node therein, as well as the index in the coefficient array at which the coefficients for that node begin.

**Table 1:** The format of the weights file

Group	Field	Type	Shape	Requirement	Description
/electrodes /{*electrodename*}	layer	utf8	1	Optional	Layer of the circuit in which {*electrodename*} is located. If the electrode is in a region without cortical layers, then “NA”. If the electrode is outside the brain, then “Outside”
/electrodes /{*electrodename*}	position	float32	3	Mandatory	Position of {*electrodename*}, in cartesian coordinates [μm]
/electrodes /{*electrodename*}	region	utf8	1	Optional	Region in which {*electrodename*} is located
/electrodes {*electrodename*}	type	utf8	1	Mandatory	Specifies the approach by which the coefficients are calculated. Takes the following values: Reciprocity, DipoleReciprocity, LineSource, or PointSource. Reciprocity refers to the equation from [6] for point sources of current. DipoleReciprocity is the simplified form of this equation for current dipole sources. LineSource means the analytic solution for current line sources in infinite, homogeneous extracellular media. PointSource refers to the analytic solution for point sources under the same simplified conditions.
/electrodes /{*electrodename*}/ {*population_name*}	electrode_id	uint64	1	Mandatory	Index of the column corresponding to this electrode in /electrodes/{population_name}/scaling_factors
/electrodes /{*population_name*}	scaling_factors	float64	Total_comp x N_elec+1	Mandatory	Scaling factor for each compartment in the corresponding neuron, in [V/nA]. All values in the last column are 1. This column corresponds to a “test electrode”. The signal from the test electrode should be ~0. If this is not the case, then the membrane currents over individual neurons do not sum to zero.
/{*population_name*}	node_ids	uint64	N_nodes	Mandatory	List of node ids. Node ids not listed here are to be ignored
/{*population_name*}	offsets	uint64	N_nodes + 1	Mandatory	The offset for each node in the scaling_factors field, followed by the index of the last element of the last node

#### 2.3.2. Electrode array file format.

The electrode array for which the coefficients are calculated is defined in a csv file. The csv file begins with the header name,x,y,z, layer,region,type. Each column of the file is described in Table 2. Each row corresponds to an additional electrode. The electrode csv file is read when the weights file is created, in order to populate the metadata.

**Table 2:** The format of the electrode csv file

Entry	Description
name	Either an index or a meaningful name describing the electrode
x	x-coordinate of the electrode (of the center of the electrode for extended electrode geometries), in μm
y	y-coordinate of the electrode (of the center of the electrode for extended electrode geometries), in μm
z	z-coordinate of the electrode (of the center of the electrode for extended electrode geometries), in μm
layer	Cytological layer in which the electrode is located, or “NA” if in a brain region without layers, or “Outside” if not within the brain. Used only to provide reference information to the user.
region	Brain region in which the electrode is located, or “Outside” if not within the brain
type	Method used to calculate the weights (Reciprocity, DipoleReciprocity, PointSource, or LineSource)

#### 2.3.3. Generation of weights files.

Discretization of the neurons into electrical compartments is performed at runtime. Therefore, to extract the number of compartments per neuron, a single-timestep simulation is run, from which a compartment report is produced. For each compartment, which are uniformly spaced, the position in Cartesian coordinates is interpolated from the 3d points in the morphology file, which do not necessarily correspond to the discretization.

After interpolation of segment positions, the H5 file defined in Sect 2.3.1 is created, with all coefficients initially set to 1. In a subsequent step, coefficients are computed, e.g., based on interpolated potential field calculated using Sim4Life (ZMT Zurich MedTech AG, Zurich, Switzerland; c.f. S1 Methods), or using the line-source approximation (c.f. Sect 2.1.3) or the point-source approximation (c.f. Sect2.1.4).

While the weights files are intended for runtime calculation of extracellular signals in the Neurodamus simulation control application (see Sect 2.3.4), it is also conceivable that other simulators, such as LFPy [19] or HNN [1], could be extended to accept BlueRecording weights files. This would allow these simulators to use the full set of extracellular signal calculation methods that BlueRecording provides, particularly the generalized reciprocity approach, which is not available in other simulators. Alternatively, tools such as LFPy could be extended to produce weights files in the format described in Table 1, which could then be used with Neurodamus (see below). As LFPy provides signal calculation methods that are not available in BlueRecording (c.f. Sect 3.1.2), this would allow those methods to be used in conjunction with the efficient neural simulation toolchain used by BlueRecording.

#### 2.3.4. Online calculation of EEG/LFP signals.

The calculation of extracellular signals is a multi-step process that begins with the launch of a Neurodamus [7] simulation. If a weights file, as defined in Sect 2.3.1, is present in the SONATA simulation config file, the weights and configuration are loaded, providing the necessary information for the subsequent extracellular signal calculation.

For each neuron distributed to a given processor, Neurodamus iterates through their corresponding sections, loading the factors associated with each section from the weights file. Neurodamus calls Neuron’s nbbcore_register_mapping function, which writes the factors to an NrnThreadMappingInfo object – a Neuron datatype which stores (among other data) the segment ids and corresponding LFP factors for each neuron on a given thread. On each thread at runtime, for each timestep CoreNEURON (the compute engine used by the NEURON simulation environment [8]) iterates through the neurons in the NrnThreadMappingInfo object, and calculates the contribution to the LFP for each neuron by summing the products of the segment transmembrane currents and their corresponding segment weights. The calculation of the contributions of the neurons to the extracellular signal is performed independently on each thread; these individual contributions are then written to a SONATA report file as described in [9]. We estimate that the online calculation of the extracellular signals adds a computational cost of 14% for 35 electrodes.

BlueRecording was developed for the CoreNEURON engine, and is not compatible with the legacy NEURON engine. Therefore, BlueRecording cannot be used in simulations of extracellular electrical stimulation or ephaptic coupling, which rely on NEURON’s extracellular mechanism, which is not supported by CoreNEURON. However, rather than modeling ephaptic coupling explicitly, it is also possible to model the effects of ephaptic coupling by injecting a current into the neuron, corresponding to the impact of the activating function. In this way, it is theoretically conceivable that BlueRecording could be used to model ephaptic coupling. The extracellular potential induced by a current from each of the *n* neural compartments would need to be calculated at the location of every other neural compartment, thus requiring ${\text{n}}^{2}$ weights to be computed. In a heterogeneous dielectric environment, this would require as many finite element simulations as there are neural compartments, which would be computationally infeasible. However, if we assume a homogeneous environment, then an analytic solution can be used to calculate the weights. It may be possible to reduce these computations to a reasonable degree of complexity using, for example, the fast multipole method. Injecting the appropriate currents into each compartment would also require the creation of a new current clamp mechanism, which would need to have access to the recorded extracellular potential. Thus, the implementation of ephaptic coupling using BlueRecording is conceivable but technically very difficult.

#### 2.3.5. Reports.

For storing simulation reports, the SONATA format [9] is utilized, which organizes the data in an HDF5 file (Table 3). This format is specifically designed to handle various types of data related to neural simulations, providing a structured and efficient way for storing and retrieving the data.

**Table 3:** The format of the simulation reports

Group	Field	Type	Requirement	Description
/report /{*population_name*}	data	float32	Mandatory	The reported values. Units is defined by the “units" attribute.
/report /{*population_name*}/ mapping	node_ids	uint64	Mandatory	The set of node ids (no duplicate).
/report /{*population_name}*/ mapping	index_pointers	uint64	Mandatory	The offset for each node in the data field.
/report /{*population_name*}/ mapping	element_ids	uint32	Mandatory	Represent the compartments as in NEURON, ordered by compartment IDs and grouped by nodes. For the ‘lfp’ report type, it represent the electrode IDs.
/report /{*population_name*}/ mapping	time	float64	Mandatory	3 values defining start time, end time, and time step. end time is not part of the report.

For extracellular reports, a unique aspect of the SONATA format is the use of the field “element ids” to represent electrode IDs, as opposed to representing compartments as in NEURON. This means that reports capture the electrical activity at specific electrode locations, rather than at individual compartments within the neural model.

An extracellular report is requested by adding an lfp block to the report section of the simulation configuration file (see [9] for a full description of this file).

"reports":{

"report_name":{

"type":"lfp",

"cells":"Mosaic",

"variable_name":"v",

"dt":0.1,

"start_time": 0.0,

"end_time": 5000.0,

"sections":"all"

}

}

The name-value pairs "type":"lfp" and "variable_name":"v" must be present verbatim; the others are user-configurable.

## 3. Results

### 3.1. Verification

#### 3.1.1. Unit tests.

Unit tests, with 70% code coverage, are provided in our Github repository, in the folder tests. These ensure, for example, that the weights files are outputted with the correct format, that segment positions are interpolated correctly for a morphology with 10 sections, and that weights are calculated correctly for a simple two-compartment model. They can be run with pytest.

#### 3.1.2. Comparison of calculation methods.

We provide a small verification example in our Github repository (https://github.com/BlueBrain/BlueRecording), comparing the generalized reciprocity approach, dipole-based reciprocity approach, line-source approximation, and point-source approximation, in a large homogeneous medium. We simulate the signal from a single Layer 5 pyramidal cell from the BBP somatosensory cortex (SSCx) model. The cell is driven with Ornstein-Uhlenbeck noise with an amplitude of 100% of the spiking threshold and a standard deviation of 1% (c.f. S2 Methods). We chose such a low standard deviation in the noise process to facilitate visualization of the differences between the signal calculation methods. In principle, a deterministic input could also have been used, but as the goal of this experiment is to verify that our calculation methods perform as expected (insofar as the differences between them match theoretical considerations), and given that Ornstein-Uhlenbeck noise input is used in larger-scale simulations described in Sects 3.2–3.4, we elect to use Ornstein-Uhlenbeck noise here as well.

**Fig 2:**
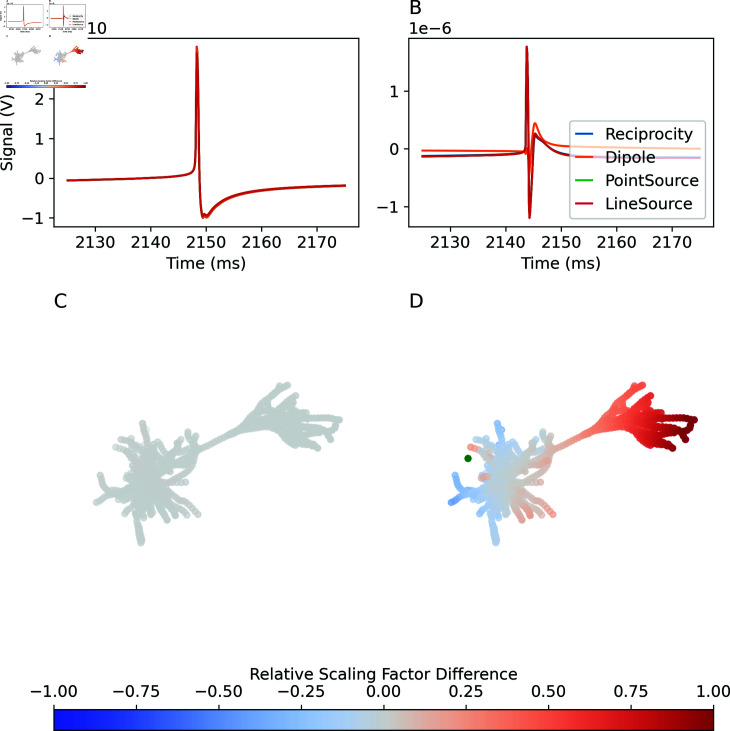
Extracellular signals recorded in a large homogeneous medium. A: Signals recorded with recording electrode (not visible in panel C) distant from the neuron. B: Signals recorded with recording electrode (green dot in panel D) near the neuron. C: Difference in per-compartment weight between generalized reciprocity and dipole-based signal calculations, for electrode far from the neuron (adjusted for constant offset, and normalized to the range of compartment weights in general reciprocity approach). D: The same, for electrode close to the neuron.

The noise input triggers an action potential in the neuron, which is observed as a large transient in the recorded signal (Fig 2A–2B). Based on theoretical considerations outlined in the introduction, we expect a weaker signal and disappearing differences between the approaches at larger distances. We confirm that when the recording and reference electrode are located far from the neuron (30 mm and 40 mm, respectively), the signals recorded using each of the methods are very similar (Fig 2A). In contrast, when the recording electrode is placed 218.5 μm from the neuron, the signals recorded using the different methods begin to diverge (Fig 2B), with the signal calculated using the dipole-reciprocity method diverging the most. This is the results of differences in the weights calculated for individual compartments (Fig 2C and 2D). For the electrode closer to the neuron, the full reciprocity approach yields more positive weights (relative to the soma) for the apical tuft and more negative weights for the basal dendrites than the simplified dipole approach. The higher polarity between tuft and basals explains the larger amplitude of the signal observed.

We further observe, as expected, that the signal recorded with distant electrodes (Fig 2A) has substantially lower amplitude than the signal recorded with nearby electrodes (Fig 2B). We do not expect that the former signal would be detectable in the presence of activity from other cells, but as the intent of this experiment is merely to compare the outputs of the different signal calculation methods, we do not consider this a limitation.

### 3.2. Biological application: Resting state EEG

We simulated a resting-state EEG, ECoG, and LFP signal originating from the Blue Brain Project reconstruction of the rat somatosensory cortex [10]. The model consists of 4.2 million biologically detailed neuron models, positioned in space according to the Paxinos Watson rat brain atlas [20], rescaled to the size of a juvenile animal. It has been shown to produce realistic firing activities [21]. In order to calculate EEG from this model, we developed a FEM model of a rat head that is spatially aligned to the BBP somatosensory cortex (c.f. S1 Methods).

**Fig 3:**
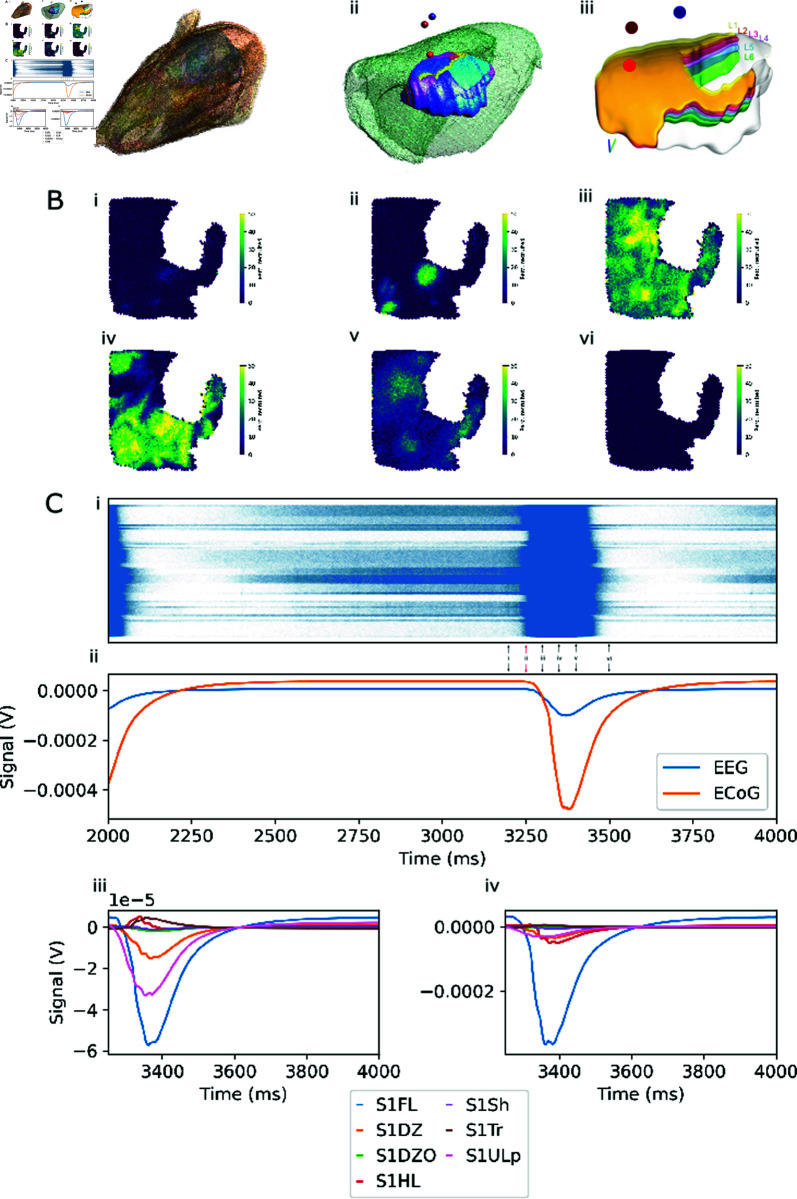
BlueRecording simulates resting-state EEG in the rat SSCx model. A. Rat head model. i: 3D view of the surface-mesh of the rat head model. ii: Comparison between the FEM model brain (green) and the BBP somatosensory cortex model. Locations of EEG electrodes over the forelimb region (dark red) and hindlimb region (blue) and ECoG electrode over the forelimb region (bright red) marked (not to scale). iii. Somatosensory cortex model, with approximate electrode locations as in ii. B: Top down view of mean firing rate over the somatosensory cortex at i. 3200 ms ii. 3250 ms iii. 3300 ms iv. 3350 ms v 3400 ms vi. 3500 ms. C: i: Raster plot of firing over the entire SSCx. Arrows indicate snapshots in panel B. Red arrow indicates start of window highlighted in panels C.iii and C.iv, and Fig 5. ii: EEG and ECoG recorded over the forelimb region. Green and red lines as in C.i. We note that due to baseline current noise injection, the signal is nonzero even in the absence of spiking activity. As the time course of recovery from hyperpolarization at the single-cell is longer than that of the action potential, we observe that the extracellular signal peak is broader than the firing burst. iii. Contributions of different regions of SSCx to the EEG recorded in ii. iv: Contributions of different regions of SSCx to the ECoG recorded in ii.

EEG is calculated from a recording electrode, positioned in the skull, directly above the forelimb region. A reference electrode is positioned over the hindlimb region. ECoG is produced by moving the recording electrode downward such that it is in contact with the cortical surface (Fig 3A). LFP is calculated by moving the electrode 1 mm into the cortex. This will place it inside cortical layer 3.

“Resting state” activity is achieved by injecting noisy conductance to neurons, which represents uncorrelated resting-state inputs from neurons extrinsic to the model (c.f. S2 Methods). The simulation is run for 5 seconds of biological time; data from the first two seconds is discarded in order to exclude an initial transient. The signal is sampled at a rate of 1 kHz. Generation of the weights file take approximately 1 hour, and the simulation runs in 7 hours on 400 nodes; each node has two 2.30 GHz, 18 core Xeon SkyLake 6140 CPUs, and 382 GB DRAM.

The model is simulated with an extracellular calcium concentration of 1.05 mM, resulting in bursts of activity over the entire somatosensory cortex (Fig 3B). Originating at discrete points, these bursts spread as travelling waves through the entire model (Fig 3B). They occur at a frequency of 0.66 Hz (Fig 3C.i), which is reflected in the EEG signal recorded over the forelimb region (Fig 3C.ii). The EEG signal is largely generated by the contribution of neurons from the forelimb region (FL) and the upper lip region (ULp), with smaller contributions from the dorsal zone (DZ) (Fig 3C.iii). Neurons in the hindlimb (HL) and trunk (Tr) area create a deflection with around a 10% of the amplitude, but opposite sign. The ECoG signal is still dominated by contributions from FL, with contributions from other regions significantly reduced, and without sign inversion in the HL and Tr regions (Fig 3C.iv).

**Fig 4:**
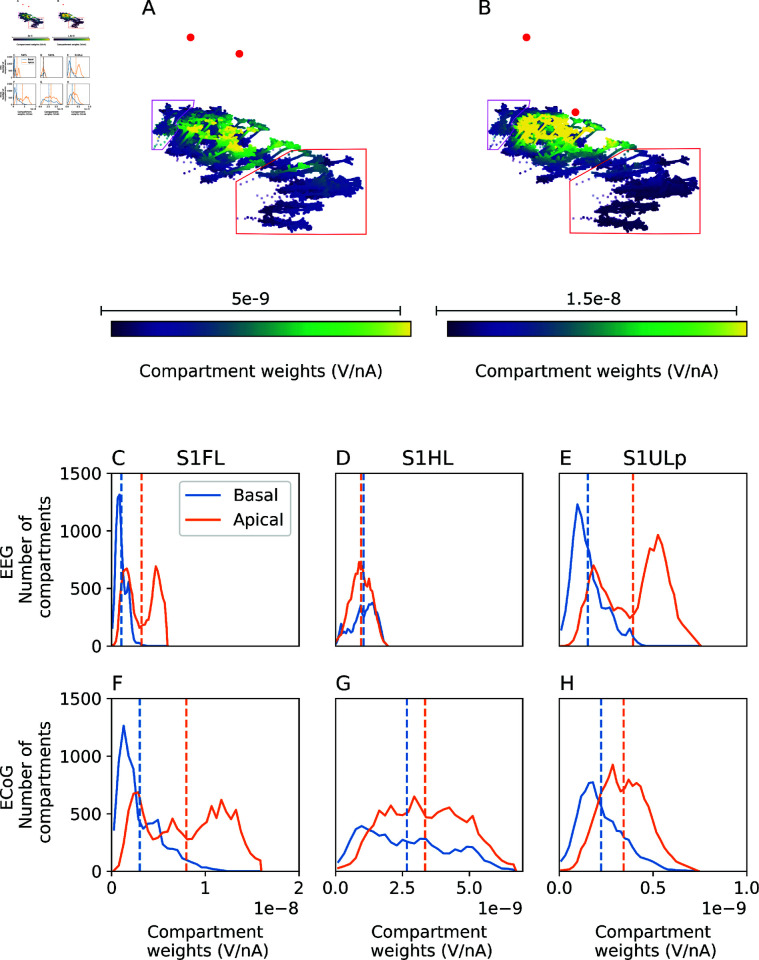
Differences in compartment weights explain differences between EEG and ECoG. A–B: Weights for EEG and ECoG recordings, respectively, calculated using the reciprocity approach, for a sample of L5 pyramidal cells in the forelimb (compartments represented as circles), hindlimb (compartments represented as triangles, enclosed in pink box) and upper lip (compartments represented as squares, enclosed in red box) regions. Electrodes are represented as red circles. Note the varying color scale ranges. C–E: Histogram of compartment weights for EEG recording, for L5 pyramidal cells in the forelimb, hindlimb, and upper lip regions, respectively. Dashed lines indicate mean values. F–H: Same as C–E, but for ECoG recording. Note that the x-axis range is different in each figure column.

To explain the difference, we investigate the weights files specifying how much the membrane current of each neuronal compartment affects the respective signal. For the EEG electrode, we found that most neurons had more positive weights associated with the apical tuft, while neurons underneath the reference electrode (corresponding to region HL) had more positive weights for their perisomatic compartments than for their tufts (Fig 4A). For ECoG, the difference in weight between apical and basal neurites is more pronounced directly under the recording electrode (corresponding to region FL), and less pronounced more laterally (corresponding to region ULp) (Fig 4B).

We confirmed this trend by considering the distribution of weights associated with basal and apical dendrite compartments (Fig 4C–4H). Specifically, apical compartments had more positive weights than basal compartments in forelimb and upper lip associated regions. For the EEG electrode, but not the ECoG electrode, basal compartments have more positive weights then apical compartments in the hindlimb region (Fig 4D vs. 4G).

If membrane currents in this highly excitable state are dominated by active currents around the soma, compensated by return currents of the apical dendrites, this explains the observed inversion between contributions from the forelimb and hindlimb regions in EEG. This also demonstrates that although the EEG and ECoG signal appear very similar under the simulated conditions, their neuronal origin is very different.

#### 3.2.1. Utility of the generalized reciprocity approach.

**Fig 5:**
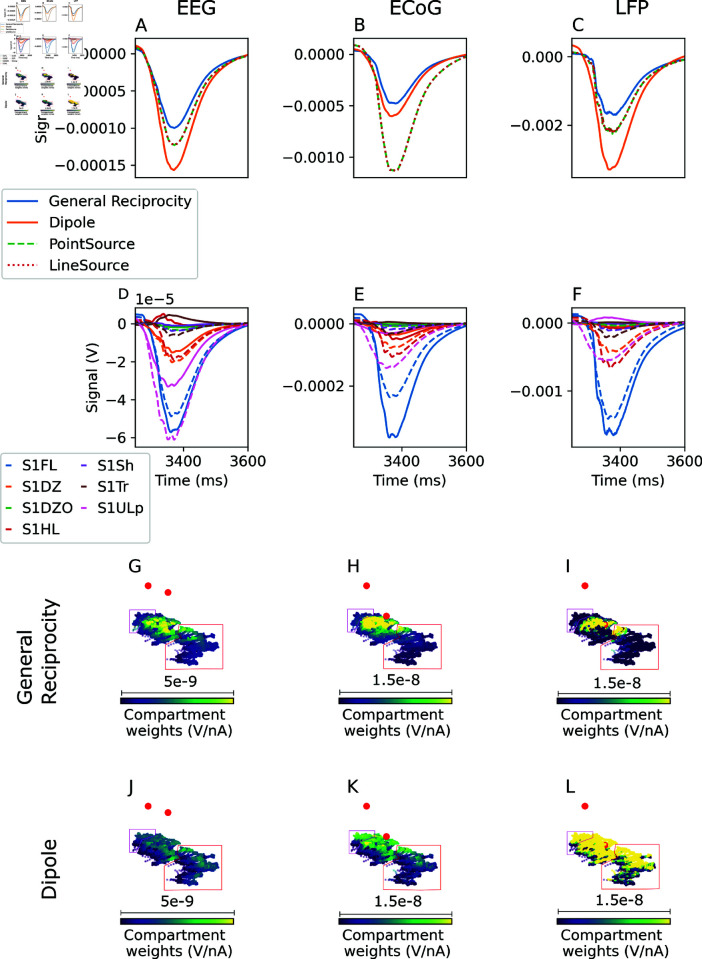
Simplified calculation methods produce inaccurate signals. EEG (A), ECoG (B), and LFP (C) signals, recorded over, or within, the somatosensory cortex, calculated with the point-source and line-source approximation, with the generalized reciprocity theorem (ground truth), and with the simplified dipole-based approaches. D-F: Contribution of each region to EEG, ECoG, and LFP signals, respectively. Solid lines indicate general reciprocity approach, dashed lines indicate dipole approach. G-I: Weights for EEG, ECoG, and LFP recordings, respectively, calculated using the reciprocity approach, for a sample of L5 pyramidal cells in the forelimb (compartments represented as circles), hindlimb (compartments represented as triangles, and enclosed in pink box) and upper lip (compartments represented as squares, and enclosed in red box) regions. Electrodes are represented as red circles. Note the varying color scale ranges. J-L: As in G-I, but calculated using the dipole approach

We compare the EEG, ECoG, and LFP signals obtained with the generalized reciprocity approach to those obtained with the dipole-based reciprocity approach and the line- and point-source approximations (Fig 5A–5C). The dipole-based approach overestimates the amplitude of the EEG and ECoG signal by a factor of ~1.5 relative to the generalized reciprocity approach (Fig 5A). This is attributable to differences in the contribution from the upper lip and hindlimb regions (Fig 5D). These differences can in turn be explained by differences between the generalized and dipole approach in the compartment weights in the upper lip and hindlimb regions (Fig 5G vs. 5J). The differences in compartment weights between the two methods arise due to the spatial heterogeneity of the E-field on the scale of the transmembrane current sources represented by a single dipole, which is accounted for by the generalized reciprocity approach (based on the finite element model of a virtual current applied between the recording electrodes), while the dipole approach assumes negligible field homogeneity , which can be inaccurate (S2 Fig). The field heterogeneity depends on the geometry of the dielectric environment.

Surprisingly, the dipole approximation provides a better fit to the generalized reciprocity signal for ECoG (Fig 5B) than for EEG. This is because, in ECoG, the dipole approximation induces a larger error in the signal contribution from the forelimb region, in the opposite direction as the error in the contribution from the other regions (Fig 5E). Thus, while the general reciprocity and dipole approaches produce similar signals, their interpretation is very different.

For LFP recordings, not only does the dipole-based approach overestimate the signal amplitude – it also changes the shape of the signal, leading to a loss of information and reduced interpretability. A smaller change in shape is also observed for the point- and line-source approximations (Fig 5C).

While the line-source and point source approaches provide a reasonable approximation of the EEG and LFP signals obtained using the generalized reciprocity approach (Figs 5A and 5C), they lead to even greater error than the dipole approximation for the ECoG signal. Therefore, the generalized reciprocity approach is the only method capable of producing realistic results in all cases.

We emphasize that the dipole reciprocity approach is derived from the generalized approach under the additional simplifying assumption that the E field is constant over the range of the neural source, which is not true near the electrode (S2 Fig). The point and line source approaches similarly involve approximations that are not required by the reciprocity-based approaches, namely, that the extracellular medium is infinite and homogeneous, and that the recording electrodes are infinitesimally small. The generalized reciprocity approach, which does not rely on such simplifications, is inherently more accurate. While the line-source approach has the benefit of accounting for the finite extent of neural segments, it is evident, e.g., from Fig 2B, that the associated error is negligible, even relatively close to neurons.

Several previous studies have investigated the adequacy of different methods for calculating extracellular signals in neural models. It has been established that, even for homogeneous tissues, within a millimeter of a neuron, the dipole approximation can produce substantial errors, but that it holds for electrode positions several millimeters from the neuron [5]. This is in accordance with our observations in Figs 2, 5C and 5F.

Naess *et al*. [5] found that in an analytically solvable four-sphere human head model, the dipole reciprocity approach performed well for EEG, but resulted in substantial errors compared to a multi-dipole approach for ECoG recordings.Halnes *et al*. [17] found that, unlike for the four-sphere human head model, the dipole reciprocity approach resulted in poor performance for EEG compared to a multi-dipole approach. In both human ECoG and mouse EEG, the dipole approximation fails when the electrode is too close to the neural current source. These findings are in accordance with our observation that in a realistic rat head model, the dipole approach results in substantial errors for both EEG and ECoG compared to the analytically correct generalized reciprocity method. Obviously, even in a realistically heterogeneous human head model, the dipole approach incurs less simplification error than in a rodent model, because of the much larger electrode-source distance. Furthermore, validation using the four-sphere human model, while advantageous because of the existence of an analytical solution, is very likely to underestimate the dipole method error: For example, scalp-to-cortex distances in real heads can vary by up to 10 mm depending on location [22] and bone thickness and composition also shows a strong variation, negatively affecting the agreement of EEG signals predicted using the dipole approximation and the ground truth [5].

Previous studies have also examined signal calculation methods that are not currently available in BlueRecording, but which may be implemented in the future. For example, Ness *et al*. [23] found that the Method of Images accurately approximates results using a forward finite element approach in a model of a microelectrode array. However, unlike reciprocity-based approaches, the Method of Images depends on highly restrictive assumptions on the structure of the dielectric medium: the neural source is assumed to be surrounded by semi-infinite homogeneous media. Beltrachini [24] showed that a finite element subtraction approach using a quadripolar neural source outperformed a similar approach using only dipoles. While this method was able to accurately approximate a distributed source for EEG recordings in a human head model, the error will necessarily be greater when the electrodes are closer to the source – which is the case for ECoG or rodent EEG – as the second-order Taylor series approximation that underlies the quadripolar approach, while superior to the lower-order dipolar approximation, will break down close to the neural source, where higher-order terms must be considered.

### 3.3. Biological application: Whisker flick

**Fig 6:**
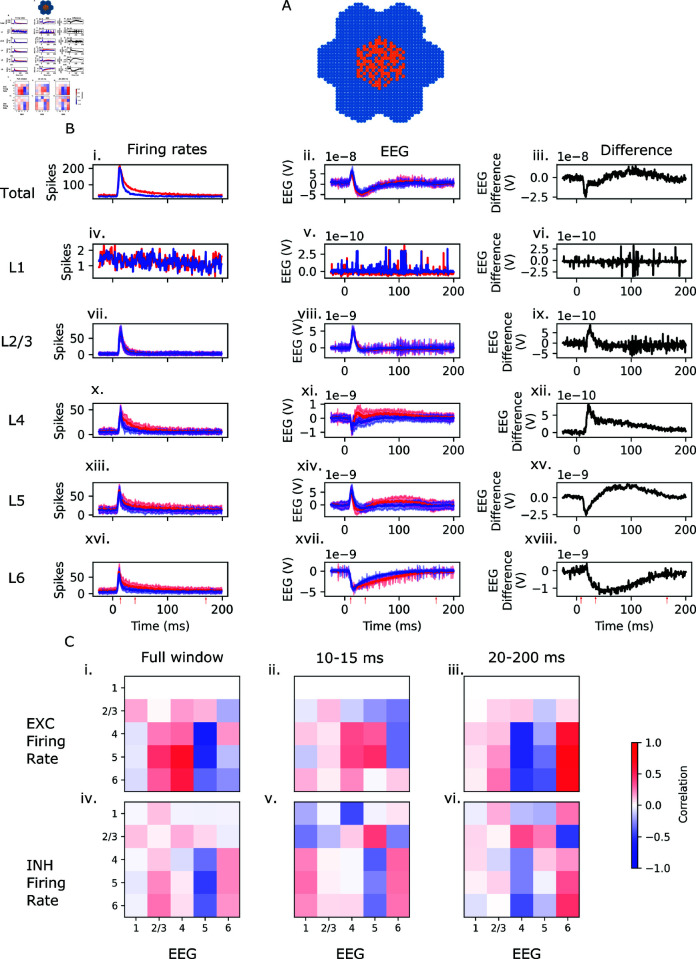
BlueRecording simulates whisker-flick EEG. A: Selected cells from the 7-column subvolume (blue) and activated thalamic projections (orange). B: Firing rate (first column) and EEG (second column) for the original and the disconnected circuit (red and blue traces, respectively) and the difference in the EEG between the two circuits (third column), for both the full circuit (first row) and each of the layers (subsequent rows). C: Correlation matrices between excitatory (first row) and inhibitory (second row) firing rates in each layer, and the differences in EEG contributions from each layer. In each correlation matrix, firing rates are represented along the rows, and EEG differences along the columns. Correlations are calculated for the full window (first column) and for windows 12-45 ms after the stimulus, and 40-200 ms after the stimulus (second, and third columns, respectively). Start and end times of the windows are marked by red arrows in panel B.

In a seven-column subvolume of the somatosensory cortex, we simulate a whisker flick stimulus, as in [21]. Briefly, a population of virtual (i.e., not biophysically modeled) thalamic fibers, projecting primarily to the central column of the subvolume (Fig 6A), is activated. We repeat the experiment for a circuit in which all cortico-cortical connections have been removed. EEG is recorded from the same electrodes used in the previous section, and averaged over 10 trials.

While stimulus triggered more sustained spiking in the connected circuits than in the disconnected circuit (Fig 6B.i), the EEG signal is remarkably similar (Fig 6B.ii). Differences are observed in the EEG contributions of different layers, particularly L4 (Fig 6B.xi), L5 (Fig 6B.xiv), and L6 (Fig 6B.xvii). However, these differences largely compensate.

We calculate the Pearson correlation between firing rates (in the original circuit) of excitatory and inhibitory cells in each layer, and the difference between the original and the disconnected circuit in the EEG contributions of cells in each layer. Because EEG is driven primarily by synaptic input [25], the correlation between the firing rate of a specified presynaptic population and the EEG difference in a specified postsynaptic population provides information about the functional connectivity between the two populations – a strong correlation between presynaptic firing rate and the difference in postsynaptic EEG suggests that the presynaptic population provides (not necessarily direct) synaptic input to the postsynaptic population. Of course, it is possible that both the firing rate of the presynaptic population and the EEG difference in the postsynaptic population are driven by a hidden third population, but this is a limitation of most functional connectivity metrics.

Over the entire time window, excitatory firing rates tend to correlate more strongly with EEG differences than inhibitory firing rates (Figs 6C.i and 6C.v). Between 12 and 40 ms after the stimulus, inhibitory firing rates in all layers besides L2/3 correlate negatively with the difference in the L5 contribution to the EEG between the original and disconnected circuit (Fig 6C.v), suggesting that the negative deflection the L5 EEG difference (Fig 6B.xv) may be due to inhibitory inputs to L5 cells that are absent in the disconnected circuit. Between 40 and 175 ms after the stimulus, there is a positive correlation between L2/3 and L4 inhibitory firing rates and the EEG difference in Layer 5 (Fig 6C.vi). This suggests that the positive deflection in the L5 EEG difference (Fig 6B.xv) may be due to inhibitory inputs from layers 2-4 to L4 that are missing in the disconnected circuit. BlueRecording thus provides a method to suggest possible interpretations of EEG signals in terms of functional connectivity, and to predict firing rates from EEG.

We do not claim that the results presented here provide general insight into the organization of cortical circuits more broadly; the extent to which the SSCx whisker flick results generalize to other regions and conditions is an empirical question beyond the scope of this paper. Rather, this study demonstrates the ability of BlueRecording to simulate neural recording *in silico* and to leverage this to disentangle and shed light on signal contributions for different subpopulations, and to elucidate the role of the functional organization of neural microcircuits. Such results can serve to formulate testable hypotheses and to constrain putative mechanisms of action. Extending this approach to other regions and regimes is a promising future application for BlueRecording.

### 3.4. Biological application: Hippocampal theta oscillations

As in [11], we simulated medial septal input to a subvolume of the hippocampal CA1 model consisting of a cylinder with diameter 600 μm. We are also able to simulate LFP recordings from the full circuit of ~456000 neurons. In both cases, we apply a depolarisation current of 120% of the spiking threshold to all cells. A sinusoidal current of -0.2 nA and frequency of 8 Hz is injected in PV+ cells using a NONSPECIFIC_CURRENT in Neuron (c.f. S2 Methods). We mimic the effect of 1.0 uM ACh on synapses and cell excitability as described in [11].

**Fig 7:**
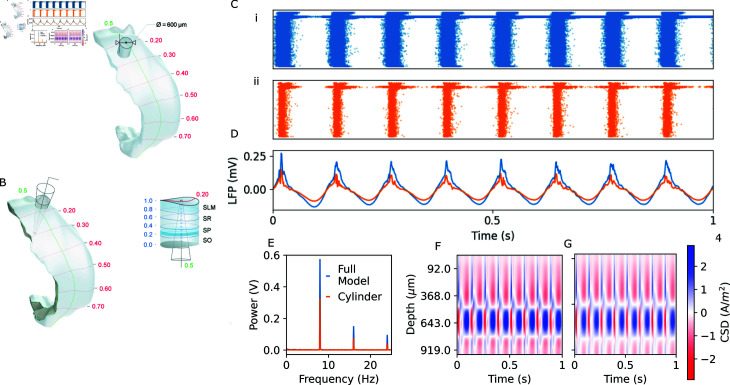
BlueRecording simulates hippocampal LFP. A: Visualization of the hippocampus and cylindrical subvolumes. B: Visualization of recording electrode placement. C: Raster plot of activity in the full hippocampus (i) and the cylindrical subvolume (ii). D: LFP recorded from a representative electrode in the hippocampus. E: Power-spectral density calculated for the signals in panel D. F: Current source density (CSD) calculated in the full hippocampus simulation. G: Current source density calculated in the cylindrical circuit. CSD maps are calculated using the standard CSD method with Vankin correction [26], as implemented by Rimehaug *et al*. [27]

We calculate the LFP signal in both circuits using BlueRecording, with the line-source approximation. We are able to replicate the finding in [11] that the simulated medial septal input results in LFP oscillations at ~ 8 Hz (Figs 7C–7G). The LFPs calculated for the two circuits (Fig 7D), as well as the resulting power-spectral densities (Figs 7E) and current-source densities (Figs 7F–7G), are very similar in both circuits, albeit with higher power in the full hippocampal circuit (Fig 7). These results suggest that the findings in [11] generalize to a larger circuit model.

### 3.5. Comparison with existing tools

In Table 4, we compare BlueRecording with several existing tools. We estimate the amount of time required by these tools to run a simulation the size of the SSCx model on equivalent hardware, under the assumption that computational time is directly proportional to network size and inversely proportional to the number of available CPU cores. Thus, for a network of size *N* cells run on *C* cores, computational time $t=\frac{t_{B}C_{B}N}{N_{B}C}$, where *t*_*B*_, *N*_*B*_, and *C*_*B*_ are reported times, network sizes, and numbers of cores reported in [28] and [29] for LFPy and Bionet, respectively. We do not account for differences in CPU clock speed or available RAM. We show that both in terms of available signal calculation methods and in terms of performance, BlueRecording outperforms the state of the art by a significant margin. However, we were unable to assess the performance of HNN, and we note that LFPy supports several analytical methods for the calculation of extracellular signals that are not supported by BlueRecording. Because the calculation of extracellular signals itself is not a significant source of computational cost, either in BlueRecording or in the alternatives described above, the performance advantages of BlueRecording can be attributed to its tight integration with CoreNEURON, which is optimized for computational performance for large networks. In contrast, neither LFPy nor BioNet use CoreNEURON. Compared to legacy Neuron, CoreNEURON speeds up computations up to 7-fold [8]. Unlike BlueRecording, where the calculation of extracellular signals is performed within CoreNEURON, both LFPy and BioNet pass membrane currents from Neuron to a Python API at each time step in order to calculate extracellular signals. Per-timestep calls to the Python interpreter are not possible in CoreNEURON. Thus, achieving the same speeds as BlueRecording in LFPy or BioNet would require significant refactoring.

**Table 4:** Comparison of BlueRecording with existing tools

Feature	Human Neocortical Neurosolver	BioNet	LFPy	BlueRecording
Line-Source Approximation	No	Yes	Yes	Yes
Analytic Calculation with Simplified Head	No	No	Yes	No
Reciprocity Calculation with Dipole Approximation	No	No	Yes	Yes
Detailed Reciprocity Calculation	No	No	No	Yes
Morphologies	Simplified morphology templates built-in; creating other morphologies nontrivial	Arbitrary	Arbitrary	Arbitrary
Circuit specification	Built-in templates; user-editable connectivity	SONATA	Ad hoc; instantiated in code	SONATA
Estimated Simulation Time for SSCx-sized Circuit (~4.2 million neurons, assuming 14400 cores)	Insufficient published data to estimate	23.3 hours	28.8 hours	7 hours

## 4. Availability and future directions

### 4.1. Code availability and dependencies

- The source code for BlueRecording, and instructions for generating the figures in Sects 3.1.2, 3.2, 3.3, and 3.4 are available at https://github.com/BlueBrain/BlueRecording.
- The seven column subvolume of the BBP circuit model is available under the following DOI: https://doi.org/10.5281/zenodo.11113043. The full BBP circuit model is available on Harvard Dataverse under the following DOI: https://doi.org/10.7910/DVN/HISHXN, and the hippocampus model is available on Harvard Dataverse under the following DOI: https://doi.org/10.7910/DVN/TN3DUI.
- Membrane mechanism files used in the neuro-simulations are available at https://github.com/BlueBrain/neurodamus-models.
- The specification for the version of the SONATA format used at BBP is available at https://github.com/BlueBrain/sonata-extension/.
- Neurodamus is available at https://github.com/BlueBrain/neurodamus.
- CoreNEURON itself is fully integrated into the NEURON simulation environment, which is available at https://github.com/neuronsimulator/nrn.
- Code for the generation of FEM models, as well as a list of dependencies, is available at https://github.com/BlueBrain/BlueBrainHeadModels. The required finite element meshes are available on Zenodo (https://doi.org/10.5281/zenodo.10926947). As this pipeline is specific to the demonstration application presented in this work, we do not consider it to be part of the core BlueRecording package.
- Finite element meshes and FEM output files used in the simulations in Sects 3.1.2, 3.2, and 3.3 are available on Zenodo (https://doi.org/10.5281/zenodo.14419388).

### 4.2. Long-term outlook

We anticipate that BlueRecording can be easily extended to permit simulation of linear signals from other recording modalities, both electromagnetic, such as MEG, and optical, such as VSDI, by simply writing the appropriate coefficients to the weights file, and in the case of VSDI, multiplying the weights by the segment voltage rather than the transmembrane current. A weights file could also be generated to directly calculate the current source density, or the current dipole itself, rather than an electromagnetic signal.

Because BlueRecording is compatible with the SONATA format, it can readily be used in hybrid models, where the dominant contributors are modeled in morphological detail, while other contributors are represented by simplified point neurons (similar to HNN) or neural masses. Such hybrid models permit the simulation of even larger models, or to run SSCx-sized models on less performant computational infrastructure.

## Supporting information

S1 Methods Methods for the generation of finite element meshes and simulation of electric fields. (PDF)

S2 Methods Methods for the input of current or conductance noise to the model. (PDF)

S1 Fig Procedure for aligning the NeuroRat head model underlying the FEM simulations with the BBP circuit model. Blue blocks represent input and output files, while pink blocks represent processes. For processes that generate transformations between two images, blue arrows represent the moving image, while red arrows represent the target image. Black arrows represent inputs that are transformed by processes and the resulting outputs. For processes that apply a transformation to an image, purple arrows represent the transformation object used.
(TIFF)

S2 Fig A. E-Field magnitude, in a cross section of the rat head, induced by current applied to an ECoG recording electrode. The scalp (beige), cancelous skull (white), and somatosensory cortex (blue) are displayed for context. B. Zoom in on the boxed area in panel a, with scalp and skull removed. Somatosensory cortex is displayed in blue. The E-field magnitude within the somatosensory cortex varies significantly (highlighted in the green box), demonstrating that the dipole approximation is not valid for ECoG recordings.
(EPS)

S1 Table The format of FEM output. We list only those fields which are used by BlueRecording. The format is identical to output files from a Sim4Life Electro-ohmic Quasi-static simulation.
(PDF)
