# Supplementary material for: BlueRecording: A pipeline for the efficient calculation of extracellular recordings in large-scale neural circuit models
Source: PLoS Comput Biol. 2025 May 23;21(5):e1013023. doi: 10.1371/journal.pcbi.1013023 (PMC12101670; doi:10.1371/journal.pcbi.1013023)
Supplement: S1 Methods — (PDF) [file pcbi.1013023.s001.pdf]

S1 Methods: Finite-element models

In order to perform the FEM electromagnetic simulations required for the reciprocity-based approach, we must generate a FEM model of the rat head that is spatially aligned to the BBP microcircuit, which is based on the Paxinos Watson atlas. We begin the process of generating the FEM model with the series of segmented MRI images underlying the ViZOO NeuroRat (150g) model V4.0 (DOI: 10.13099/VIP91106-04-1, https://itis.swiss/virtual-population/animal-models/animals/neurorat/). The procedure used for aligning the NeuroRat model to the BBP circuit model is outlined in S1 Fig.

The NeuroRat head model is segmented according to the Waxholm atlas [1]. As this brain segmentation is relatively coarse, we re-segment the NeuroRat brain with labels from the SIGMA atlas [2]. In order to do so, we create a version of the SIGMA label map, with each region assigned to the corresponding coarser labels from the Waxholm atlas. An affine transformation between the relabeled SIGMA label field and the Waxholm atlas is calculated using the FSL FLIRT tool with a label-difference metric [3]. This transform is applied to the original SIGMA model, yielding a model that is aligned to the Waxholm atlas but with SIGMA labels.

A nonlinear transformation aligning the Waxholm atlas to the NeuroRat is calculated with Advanced Normalization Tools (ANTs). This transforamtion is applied to the Waxholm atlas but with SIGMA labels, yielding a model aligned to the NeuroRat, but with SIGMA labels. The SIGMA labels are assigned to the corresponding labels in the Paxinos Watson atlas, and an affine transform is calculated, as before, from the NeuroRat brain to a digitization of the Paxinos Watson atlas created, as described in Supplementary Materials A of [4], by aligning, rasterizing and interpolating the individual slices obtained from the CD-ROM distributed with [5]. This transformation is then applied to the full NeuroRat label field (cropped to include just the head), to produce a rat head model aligned to the Paxinos Watson atlas. A scaling factor of 0.96837 is then applied to account for the difference between the adult and juvenile rat.

Finally, the label field is transformed into a discretized mesh (Fig 3A.i) using Sim4Life (ZMT Zurich MedTech AG). The mesh positioning is manually adjusted to ensure that the upper layers of the SSCx does not extend into the cerebrospinal fluid. This procedure resulted in a good match between the Paxinos Watson brain and the FEM head model (Fig 3A.ii). Recording electrodes, modeled as small spheres, are positioned on the head model as described in Section 3.2.

FEM simulations were executed using the Electro-Ohmic Quasi-Static solver in Sim4Life, which solves the equation $\nabla\sigma\nabla\phi = 0$, where σ is the electrical conductivity and ϕ is the electric potential, from which the electric field can be obtained as $E = -\nabla\phi$. This quasi-static approximation of Maxwell's equations can be used, because at the frequencies of interest, displacement currents are negligible compared to ohmic currents and the domain is much smaller than the wavelength [6]. Tissue properties were assigned in accordance with the low-frequency dielectric properties from the IT'IS Tissue Properties Database V4.1 [7]. All brain regions outside of the cerebellum and brainstem are assigned a conductivity of ~0.37S/m, and both cortical and cancelous skull are assigned a conductivity of ~0.018S/m. To determine the electric potentials required for the application of the general form of the reciprocity theorem, Dirichlet boundary conditions were applied to one of the recording electrodes (1 V) and the reference electrode (-1 V). The applied total current is calculated by integrating the normal component of the current flux density $j = \sigma E$ over a closed surface surrounding the recording electrode, but excluding the reference electrode. The cortex is discretized at a resolution of 0.2 mm, the skull at a resolution of 0.4 mm; the electrodes are discretized using the Sim4Life Automatic Grid at Extremely Fine resolution, while

other tissues are discretized at Default resolution. This results in a mesh of ~30 MCells, with a minimum resolution of ~20μm and a maximum resolution of ~1cm. The solver convergence settings are set to a relative tolerance of 1e-12, an absolute tolerance of 1e-50, a divergence value of 1e50, and a iteration number maximum of 100000.

References

1. Papp EA, Leergaard TB, Calabrese E, Johnson GA, Bjaalie JG. Waxholm Space atlas of the Sprague Dawley rat brain. Neuroimage. 2014;97:374–386.

2. Barrière DA, Magalhães R, Novais A, Marques P, Selingue E, Geffroy F, et al. The SIGMA rat brain templates and atlases for multimodal MRI data analysis and visualization. Nat Commun. 2019;10(1):5699.

3. Jenkinson M, Beckmann CF, Behrens TE, Woolrich MW, Smith SM. FSL. Neuroimage. 2012;62(2):782–790.

4. Bolaños-Puchet S, Teska A, Hernando JB, Lu H, Romani A, Schürmann F, et al. Enhancement of brain atlases with laminar coordinate systems: Flatmaps and barrel column annotations. bioRxiv. 2024;doi:10.1101/2023.08.24.554204.

5. Paxinos G, Watson C. The rat brain in stereotaxic coordinates (6th ed). Academic Press/Elsevier; 2007.

6. Gratiy SL, Halnes G, Denman D, Hawrylycz MJ, Koch C, Einevoll GT, et al. From Maxwell's equations to the theory of current-source density analysis. European Journal of Neuroscience. 2017;45(8):1013–1023. doi:10.1111/ejn.13534.

7. Hasgall PA, Di Gennaro F, Baumgartner C, Neufeld E, Lloyd B, Gosselin MC, et al.. IT'IS Database for thermal and electromagnetic parameters of biological tissues; 2018. Available from: itis.swiss/database.
