# Supplementary material for: BlueRecording: A pipeline for the efficient calculation of extracellular recordings in large-scale neural circuit models
Source: PLoS Comput Biol. 2025 May 23;21(5):e1013023. doi: 10.1371/journal.pcbi.1013023 (PMC12101670; doi:10.1371/journal.pcbi.1013023)
Supplement: S2 Methods — (PDF) [file pcbi.1013023.s002.pdf]

S2 Methods: Noise input

In [1], noisy conductance was injected into the neurons of the SSCx model in order to represent uncorrelated synaptic input. This was accomplished in Neurodamus using the `SEClamp` mechanism built into neuron, which models a voltage clamp with variable input resistance. For numerical reasons, the currents injected by the `SEClamp` are calculated at different time points than the transmembrane currents, meaning that total currents do not obey Kirchoff's law, leading to inaccurate calculations of extracellular signals. We therefore created a new mechanism, `conductanceSource`, which is identical to `SEClamp`, but which is treated by CORENEURON as an ion channel current rather than an electrode current. By using `conductanceSource` instead of `SEClamp` to inject noise, the input current can be accounted for in the calculation of the extracellular signals. Similarly, we add a `MembraneCurrentSource` mechanism to replace the Neuron builtin `IClamp` current clamp.

As there may be cases in which a user wishes to use `SEClamp` or `IClamp` to model a physical voltage clamp rather than a synaptic input, we add the key `represents_physical_electrode` to the SONATA simulation configuration file for all noise sources. If this key is set to True, the `SEClamp` or `IClamp` mechanism is used, for conductance and current sources, respectively. In these cases the injected current will not be accounted for in the calculation of the extracellular signal. For example, the relevant block of the SONATA simulation configuration file for a physical electrode injecting noisy conductance might read as follows

```
"Stimulus_gExc_L23E":{
  "input_type":  "conductance"
  "module":  "relative_ornstein_uhlenbeck",
  "delay":  0,
  "duration":  3000,
  "reversal":  0,
  "tau":  2.7,
  "mean_percent":  17.918,
  "sd_percent":  7.167,
  "node_set":  "Layer23Excitatory",
  "represents_physical_electrode":true
}
```

References

1. Isbister JB, Ecker A, Pokorny C, Bolaños-Puchet S, Santander DE, Arnaudon A, et al. Modeling and Simulation of Neocortical Micro- and Mesocircuitry. Part II: Physiology and Experimentation. bioRxiv. 2023;doi:10.1101/2023.05.17.541168.
