# Supplementary figures and images for: BlueRecording: A pipeline for the efficient calculation of extracellular recordings in large-scale neural circuit models
Source: PLoS Comput Biol. 2025 May 23;21(5):e1013023. doi: 10.1371/journal.pcbi.1013023 (PMC12101670; doi:10.1371/journal.pcbi.1013023)

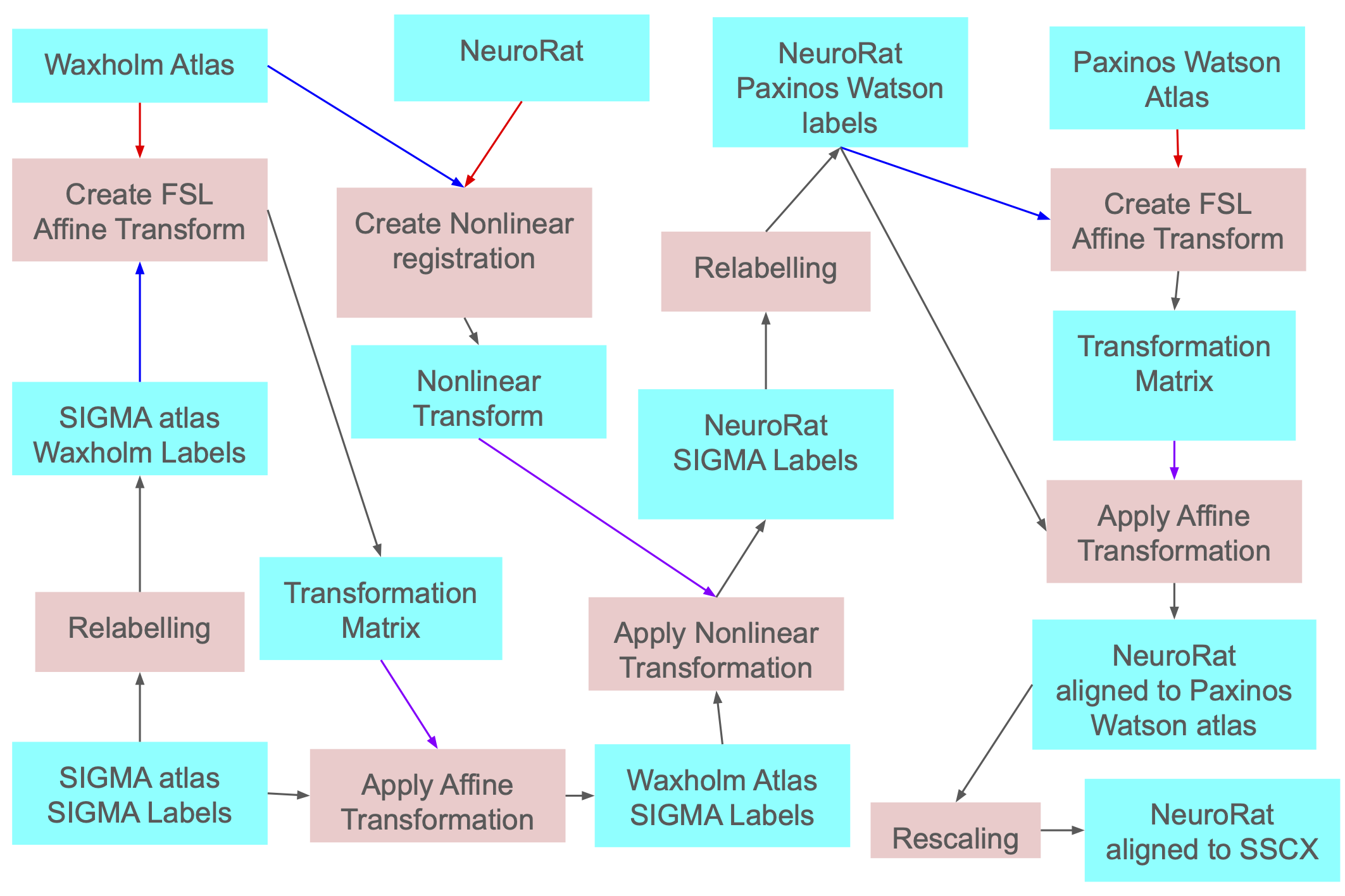

Supplement: S1 Fig — Blue blocks represent input and output files, while pink blocks represent processes. For processes that generate transformations between two images, blue arrows represent the moving image, while red arrows represent the target image. Black arrows represent inputs that are transformed by processes and the resulting outputs. For processes that apply a transformation to an image, purple arrows represent the transformation object used. (TIFF) [file pcbi.1013023.s003.tiff]
