# Supplementary material for: BlueRecording: A pipeline for the efficient calculation of extracellular recordings in large-scale neural circuit models
Source: PLoS Comput Biol. 2025 May 23;21(5):e1013023. doi: 10.1371/journal.pcbi.1013023 (PMC12101670; doi:10.1371/journal.pcbi.1013023)
Supplement: S1 Table — The format is identical to output files from a Sim4Life Electro-ohmic Quasi-static simulation. (PDF) [file pcbi.1013023.s005.pdf]

Group	Field	Size	Units	Description
/Meshes/{*mesh_ id*}	axis_x	n_x	m	x edges of mesh
/Meshes/{*mesh_ id*}	axis_y	n_y	m	y edges of mesh
/Meshes/{*mesh_ id*}	axis_z	n_z	m	z edges of mesh
/FieldGroups/{*simulation_ id*}/AllFields /EM E(x,y,z,f0)/_Object/Snapshots/0	comp0	$n_x - 1 \times n_y \times n_z$	V/m	The x-component of the E field
/FieldGroups/{*simulation_ id*}/AllFields /EM E(x,y,z,f0)/_Object/ Snapshots/0	comp1	$n_x \times n_y - 1 \times n_z$	V/m	The y-component of the E field
/FieldGroups/{*simulation_ id*}/AllFields /EM E(x,y,z,f0)/_Object/Snapshots/0	comp2	$n_x \times n_y \times n_z - 1$	V/m	The z-component of the E field
/FieldGroups/{*simulation_ id*}/AllFields /EM Potential(x,y,z,f0) /_Object/Snapshots/0	comp0	$n_x \times n_y \times n_z$	V	The electric potential
/	CurrentApplied	1	nA	Current applied between recording electrodes
